# Chlorine-UV hybrid disinfection: a review on mechanisms and efficiency towards emerging pollutants

**DOI:** 10.1007/s11356-026-37467-8

**Published:** 2026-02-11

**Authors:** Sandeep Singh Shekhawat, Rahul Kumar Goswami, Akhilendra Bhushan Gupta, Faizal Bux

**Affiliations:** 1 Institute for Water and Wastewater Technology, Durban University of Technology, Durban, 4001 South Africa; 2 Department of Civil Engineering, Malaviya National Institute of Technology, Jaipur, India

**Keywords:** Antibiotic-resistant bacteria, Antibiotic resistance genes, Disinfection byproducts, Microbial regrowth, Reactive oxygen species, Reactive chlorine species

## Abstract

This review comprehensively evaluates the direct and indirect disinfection mechanisms and performance of chlorine-UV hybrid disinfection (CUV-HD) for the removal of microbial and emerging contaminants, including post-disinfection regrowth and formation potential of disinfection byproducts (DBPs), to ensure safe reuse of treated effluents. Direct disinfection reactions are mediated by HOCl and OCl⁻, as well as UV-induced photolytic damage. In contrast, indirect inactivation is driven by reactive oxygen species (ROS), particularly ∙OH, and reactive chlorine species (RCS) such as Cl∙, ClO∙, and Cl₂∙⁻. The order of reactive species generation indicates UV/chlorine > chlorine–UV > UV–chlorine, which is supported by the effective reduction potential and, consequently, the disinfection efficacy against microbial pollutants in treated wastewater. However, the water matrix, including pH, temperature, organic matter, and total suspended solids (TSS), significantly influences both the disinfection efficiency of these hybrid strategies and the potential formation of disinfection byproducts (DBPs). The formation potential of DBPs follows the order: UV/chlorine > UV–chlorine > chlorine–UV. Further studies quantifying reactive chlorine species (RCS) and elucidating their roles in CUV-HD are needed to fully understand the underlying indirect disinfection mechanisms.

## Introduction

Wastewater treatment and reuse are sustainable approaches increasingly adopted to address rising water demand and scarcity (Kesari et al. 2021; Obaideen et al. 2022). Most treated wastewater is used for non-potable purposes, particularly in agricultural and landscape irrigation, which are among the major water-consuming sectors. Reuse of inadequately treated wastewater is linked to bacterial, parasitic, and viral infections (Adegoke et al. 2018; Kesari et al. 2021). The survival of microbial contaminants such as antimicrobial-resistant bacteria (AMR), antibiotic resistance genes (ARGs), and OPs in treated wastewater poses serious public and environmental health challenges for wastewater treatment technologies (Kesari et al. 2021; Makuwa et al. 2023). The emergence of AMR in wastewater treatment plants (WWTPs) and its disposed ecosystems such as seawater, rivers, or irrigated soils has been increasingly reported (Singh et al. 2022; Honda et al. 2023; Della-Negra et al. 2025).

Many studies have shown that the final reduction of microbial populations at WWTP is less than required by national or global regulatory authorities (Bailey et al. 2018; Igere et al. 2020; Omohwovo 2024). Moreover, merely meeting the norms of faecal indicator bacteria (FIB), traditionally *Escherichia coli* (*E. coli*) or faecal coliforms in treated effluent, does not ensure the absence of emerging pathogens due to the poor correlation between FIB and pathogens and viruses (Harwood et al. 2005; Bailey et al. 2018; Devane et al. 2020). Exposure to FIB and pathogens in treated wastewater can cause a range of health concerns such as gastroenteritis, cholera, schistosomiasis, environmental enteropathy, and stunting, particularly among farm workers and communities in close contact with reclaimed water (Poopedi et al. 2023). Secondary and tertiary treatment of WWTPs effectively reduces a large proportion of microbial populations. However, the disinfection step in tertiary treatment remains critical for effectively removing the microbial contamination.

Disinfection is the final step of water and wastewater treatment technology to control the microbial risks to human and environmental health (Zhong et al. 2019; Obayomi et al. 2024). Chlorination, UV irradiation, and ozonation are the most widely used disinfection strategies in WWTPs (Koseoglu-Imer et al. 2023). The persistence of certain pathogens, such as *Cryptosporidium*, *Giardia*, and various bacteria and viruses, in post-chlorinated water has raised concerns regarding the effectiveness of chlorine disinfection (Harwood et al. 2005; Kong et al. 2021). Additionally, sub inhibitory concentrations of chlorine can induce horizontal gene transfer (HGT) and promote the dissemination of antibiotic resistance within the microbial community in water and wastewater (Wang et al. 2020). Moreover, chlorine has a high potential for forming disinfection byproducts (DBPs) in wastewater, raising concerns about the safety of treated wastewater reuse, as some DBPs are potentially carcinogenic (Hua and Reckhow 2007; Verma et al. 2017; Forster et al. 2025). In contrast, UV irradiation has increasingly replaced chlorine in many developed nations, driven by innovations that have made it more cost-effective, environmentally friendly, and available as a compact technology. UV is a physical disinfectant that is highly effective against chlorine-tolerant pathogens and produces negligible amounts of DBPs, making it a promising alternative for wastewater disinfection (Kong et al. 2021; Koseoglu-Imer et al. 2023).

In recent decades, the increasing generation of wastewater has been reported to be accompanied by the widespread emergence of antibiotic-resistant bacteria (ARB), ARGs, and opportunistic pathogens (OPs) (Nasrollahi et al. 2022). Although prevailing standalone disinfection practices are regularly conducted following microbial indicator standards for treated water reuse, their effectiveness is limited against such emerging microbes. Furthermore, post-disinfection microbial regrowth has also become a significant concern for public and environmental health (Gupta et al. 2022). Therefore, current disinfection practices using standalone disinfectants raise concerns about their effectiveness against emerging microbial pollution, as well as the adequacy of microbial indicator assessments.

In recent years, CUV-HD disinfection approaches have been investigated to address the limitations of standalone chlorine, UV, and ozone disinfection technologies (Wang et al. 2020; Shi et al. 2021; Shekhawat et al. 2021). These synergistic or hybrid disinfection processes offer substantial benefits by addressing the weaknesses of single disinfection methods, thereby improving disinfection reliability and operational flexibility. Many studies have reported the effectiveness of CUV-HD approaches such as sequential chlorine and UV (chlorine-UV), UV and chlorine (UV-chlorine), and simultaneous chlorine and UV (chlorine/UV) in removing microbial indicators, emerging pollutants including ARB, ARGs, and OPs, as well as suppression of post-disinfection regrowth (Rattanakul et al. 2015; Destiani and Templeton, 2019; Shi et al. 2021). It is important to further understand and evaluate the disinfection efficacy of different CUV-HD approaches for emerging pollutants in different wastewater treatment settings such as hospitals, municipal, industrial wastewater, and their receiving environments to ensure safe reuse of treated wastewater.

To address increasing water scarcity and safe reuse of treated wastewater, the upgrade and reconstruction of WWTPs should be integrated with suitable hybrid disinfection technology. Each WWTP must implement the hybrid disinfection technology that is most effective and reliable in tackling the specific target pollutants and reducing risks pertinent to its wastewater characteristics and operational settings. This review provides comparative mechanistic insights and assesses the efficacy of CUV-HD approaches by elucidating their direct and indirect disinfection mechanisms for the removal of microbial pollutants, including pathogens, ARB, ARGs, and DBPs. It further highlights their effectiveness in preventing post-disinfection regrowth in treated effluents and discusses the major challenges associated with exploiting indirect disinfection mechanisms, particularly due to the limited understanding of the reactive species formed and their precise role in disinfection during CUV-HD processes.

## Disinfection technologies for wastewater treatment

**Fig. 1:**
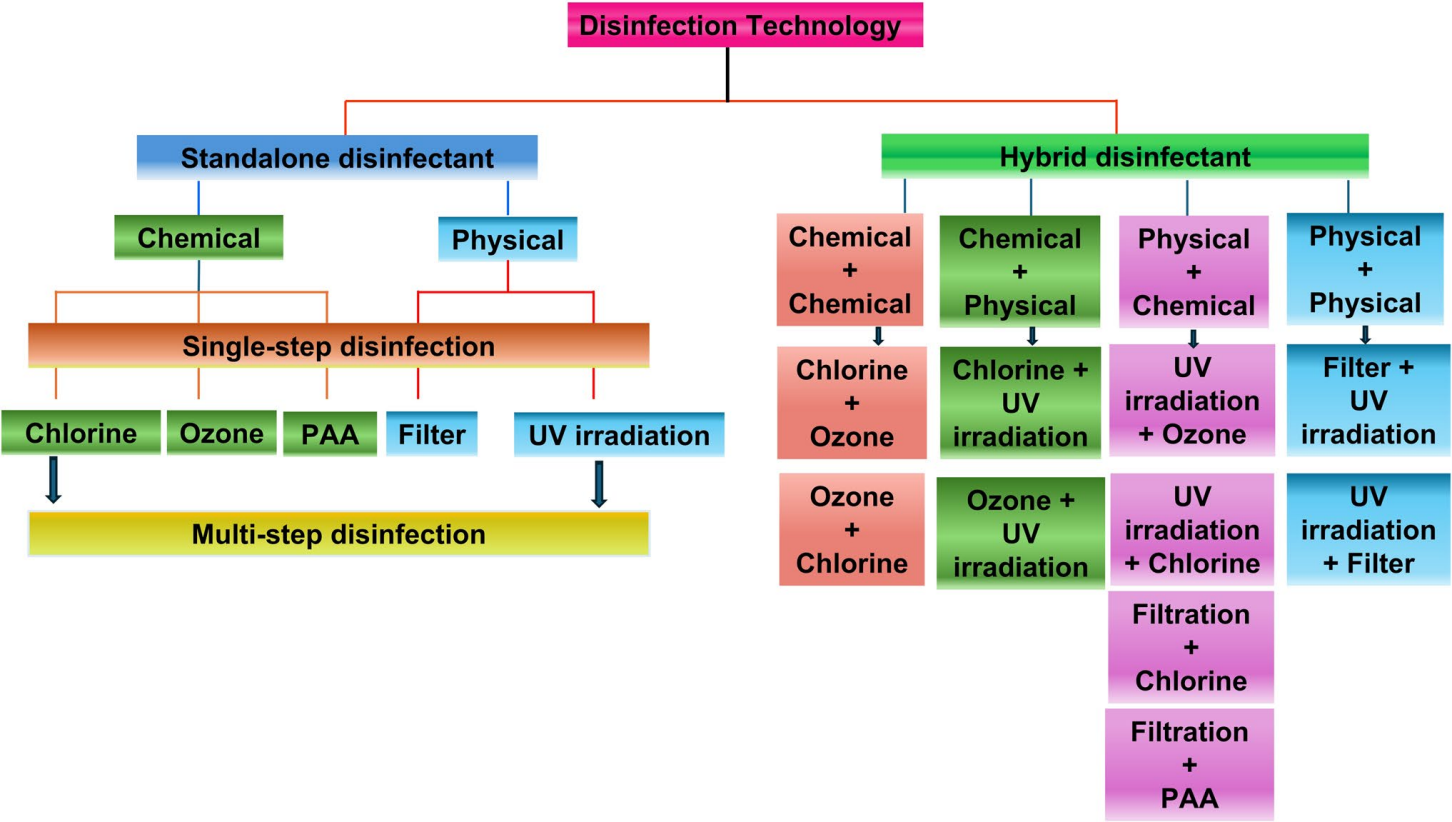
Schematic classification of disinfection technologies

Disinfection technologies are broadly classified into two categories based on the nature of the process: chemical disinfection and physical disinfection. However, biological disinfection has not yet been established as a method for water treatment. In the context of recent research and field applications in water and wastewater disinfection, research and field practices ranging from small to large-scale applications, disinfection technologies are categorized based on their application strategies into standalone and hybrid systems, as illustrated in Fig. 1.

## Disinfection mechanisms

### Standalone disinfection technologies

Standalone disinfectants such as chlorine, UV irradiation, and ozone inactivate the bacteria and other microbial contaminants by direct and indirect mechanisms (Magbanua Jr. et al. 2006; Kong et al. 2021; Shi et al. 2021). UV irradiation is more pronounced for direct inactivation of microbes by attacking directly to genetic material, thereby preventing its replication and further expression. The UV-C spectrum (200–280 nm) has been extensively used for its germicidal action against microbial pathogens. Low-pressure UV-C lamps, which emit predominantly at 253.7 nm, represent the standard germicidal wavelength and are widely applied for drinking water and wastewater disinfection (Augsburger et al. 2021). UV light-emitting diodes (UV-LEDs) that produce wavelengths in the 260–270 nm range are increasingly being investigated as a cost-effective alternative for UV disinfection (Itani and El Fadel 2023). In contrast, medium-pressure UV lamps emit across a broad spectrum of the UV-C range and have also been explored for water disinfection, providing higher fluence rates but less spectral selectivity compared to low-pressure lamps (Augsburger et al. 2021). UV-C targets key microbial biomolecules, specifically DNA, RNA, and proteins, damaging the genomic system by inducing pyrimidine dimers, nucleic acid lesions, and protein disruptions, without necessarily killing the cells (Raeiszadeh and Adeli 2020). USEPA (2022) and NSF/ANSI (2024) recommend a UV dose of 40 mJ/cm^2^ for water disinfection, which is sufficient to achieve up to a 4-log reduction of microbial pathogens. UV irradiation is safe in terms of DBP formation but does not provide a residual effect, unlike chlorine (Liu and Hu 2020; Shi et al. 2021). UV is generally effective at degrading large DNA/RNA fragments, but it is less efficient at degrading short DNA fragments, such as ARGs and mobile genetic elements (MGEs) (Liu and Hu 2020). Moreover, microbial regrowth post UV disinfection leads to UV-resistant microorganisms through photo-reactivation (Cao et al. 2025).

Both chlorine and ozone act as electrophilic oxidants in their direct disinfection mechanisms, targeting electron-rich components of microbial cells. In chlorine disinfection, chlorine reacts with water to form hypochlorous acid (HOCl), which further dissociates into hypochlorite ion (OCl⁻) (Mehendale et al. 2023). HOCl is a much stronger (80–100X) germicidal agent than OCl⁻, and the pH of wastewater strongly influences speciation; at pH values > 7–8, OCl⁻ becomes dominant, reducing overall disinfection efficiency (Mehendale et al. 2023). HOCl, especially in its un-ionized form, readily penetrates microbial cell walls and disrupts cell integrity by causing leakage of cellular components. Both HOCl and OCl⁻ are classified as free available chlorine (FAC), which, in addition to microbial inactivation, also oxidizes organic and inorganic pollutants; however, this process can generate harmful DBPs, especially under high chlorine doses or in the presence of natural organic matter (NOM) (Clayton et al. 2021). In step chlorination, either two-step or three-step chlorine dosing is applied to enhance disinfection efficiency while reducing the overall chlorine concentration (Li et al. 2017a, b; Islami et al. 2019). This approach exploits the higher microbial removal efficiency achieved during the initial short contact period (5–10 min) with a lower chlorine dose (0.5–2 mg/L), which is then reapplied in subsequent steps to form a multi-step chlorination process. Moreover, step chlorination has been reported to lower the formation potential of DBPs (Islami et al. 2019; Li et al. 2017b). However, post-treatment microbial regrowth following multi-step chlorination has not yet been studied, and it represents a critical knowledge gap. The enhanced microbial reduction efficiency and lower DBP formation potential may result from the repeated application of chlorine at short contact times, which effectively targets microbial indicators due to their high sensitivity while limiting DBP generation.

### Hybrid disinfection technologies

**Fig. 2:**
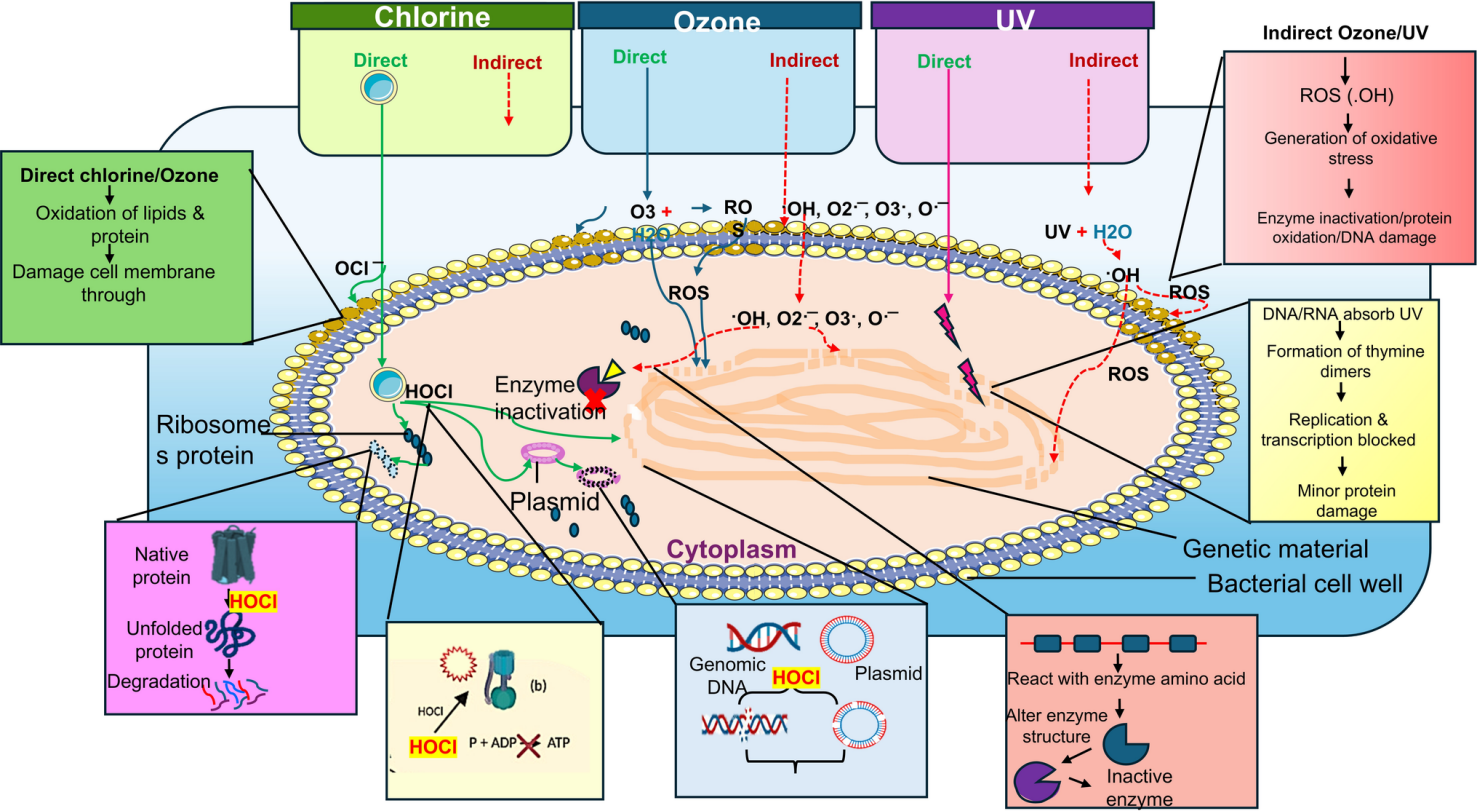
Standalone chlorine, UV, and ozone disinfection mechanisms representation

CUV-HD technologies generally represent the sequential and simultaneous application of two or more disinfectants to control the microbial contaminants in water and wastewater (Magbanua Jr. et al. 2006; Shi et al. 2021; Ping et al. 2022). These approaches aim to reduce chlorine dose and achieve a synergistic effect between the two disinfectants, enhancing the inactivation of pathogens, including ARB and ARGs (Wang et al. 2012, 2020; Zhang et al. 2015). CUV-HD approaches using chlorine and UV can be applied in three main strategies: sequential chlorine followed by UV (chlorine–UV), sequential UV followed by chlorine (UV–chlorine), and simultaneous application of chlorine and UV (chlorine/UV), to evaluate their potential for removing microbial contaminants. Other combined approaches, such as chlorine with para-acetic acid (PAA) or ozone with UV, have also been studied and can be categorized as chemical or physical disinfection presented in Fig. 1. It is important to understand the application process of the two disinfectants, produced reactive species, and available residual effects, and how these collectively play a role in the direct and indirect mechanism performed to inactivate the microbial pollutants in water during three CUV-HD approaches (Fig. 2).

#### UV–chlorine disinfection (UV-chlorine)

**Fig. 3:**
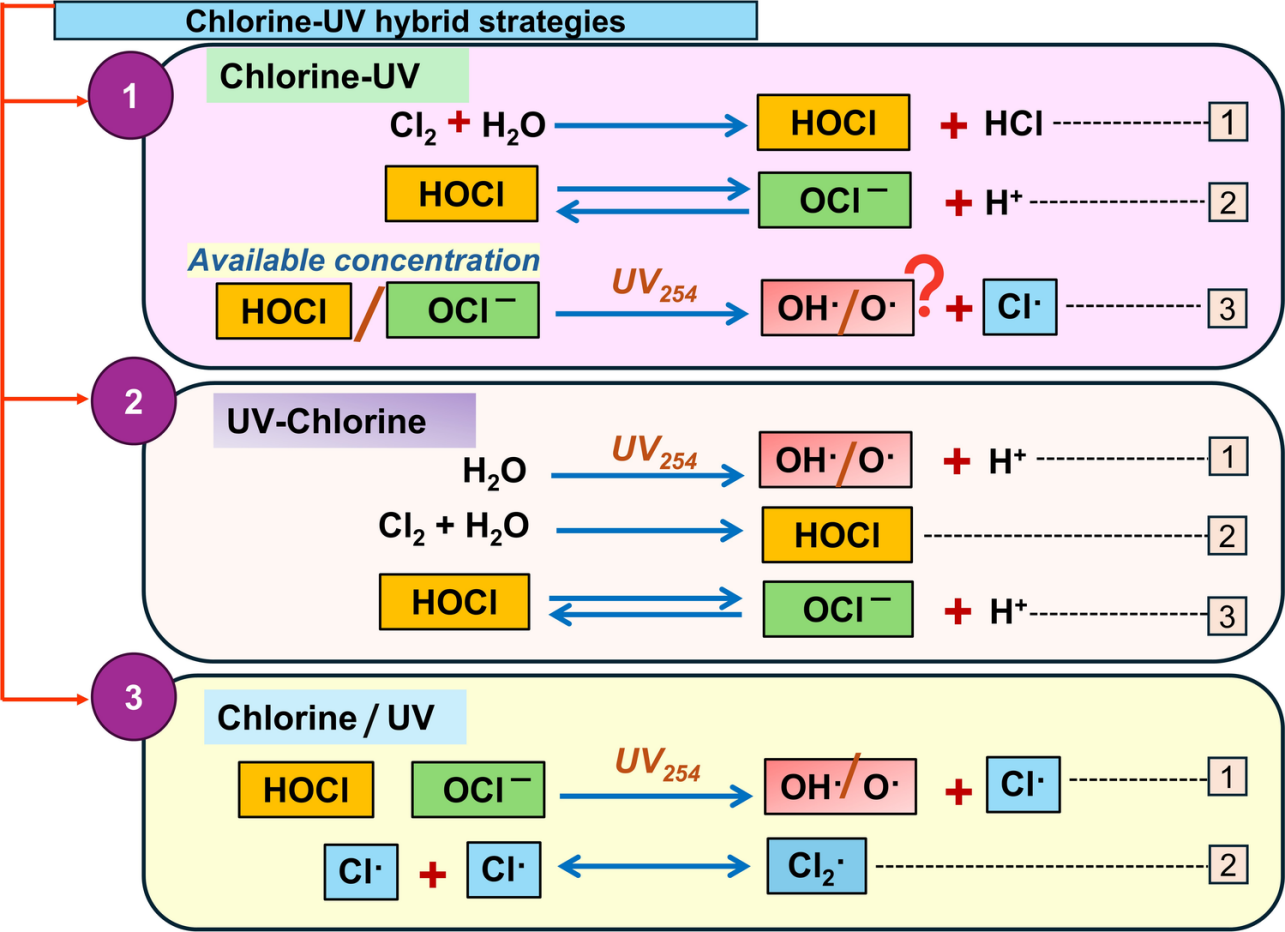
Representation of free chlorine species and reactive species in CUV-HD strategies

In this disinfection strategy, as presented in Fig. 3, the contribution of ROS and RCS is relatively low due to the limited or no availability of chlorine for UV photolysis, resulting in negligible formation of RCS (Guo et al. 2022). The disinfection mechanism involves an initial UV irradiation step, during which ROS such as hydroxyl radical (∙OH) and singlet oxygen (^1^O₂) are generated (Wan et al. 2020). These species target microbial cellular biomolecules and ARGs (Wang et al. 2024; Zhang et al. 2025). In addition, UV light is directly absorbed by the microbial genome, leading to the formation of pyrimidine (thymine-thymine) dimers that block DNA replication and also degrade large DNA fragments (Liu and Hu 2020). When chlorine is added afterward, it oxidizes pre-damaged cells and extracellular DNA (eDNA) more efficiently at lower doses.

UV-chlorine is therefore effective in ARGs and eDNA degradation since chlorine reacts with already UV-altered organics at lower concentrations (Wang et al. 2012, 2023; Lu et al. 2022; Chen et al. 2023). Although this approach is limited in exploiting indirect disinfection due to the insufficient generation of reactive species, its effectiveness can also be reduced in waters with high turbidity or suspended solids, which shield microbes from UV exposure (Liang et al. 2025). Consequently, the disinfection primarily occurs through direct inactivation. This approach is primarily applied to reduce chlorine doses and control post-disinfection regrowth, as UV treatment sensitizes microbes, making them more susceptible to inactivation at lower chlorine doses (Wang et al. 2020, 2012). Additionally, it provides a residual chlorine effect in stored water, helping maintain safety over time.

Its comparative analysis with two other hybrid disinfection strategies shows a more effective removal of viruses, chlorine-tolerant, and UV-resistant bacteria. Further, it has proven better for controlling post-disinfection regrowth, compared to standalone disinfectants and UV/chlorine hybrid disinfection (Rattanakul et al. 2015; Zyara et al. 2016; Shekhawat et al. 2023). However, it is less recommended than the other two approaches for the efficient removal of emerging microbial pollutants such as ARGs, ARBs, DBPs, and other potential microbial pathogens (Zhang et al. 2025; Chen et al. 2023). However, it is more sensitive to the water matrix such as total suspended solids (TSS) and organic matters (Wang et al. 2023).

#### Chlorine-UV disinfection (chlorine-UV)

In this strategy, as given in Fig. 3, chlorine is first applied to water, where it dissociates into HOCl and OCl^−^, remaining as free residual chlorine (Mehendale et al. 2023). When UV irradiation is applied (generally after 5 to 30 min), it photolyzes the remaining HOCl and OCl^−^, forming ROS (∙OH) and RCS (Cl∙, Cl_2_∙-, and ClO∙^–^) (Guo et al. 2022; Wang et al. 2024). The generation of these species is affected by chlorine dose, UV dose, UV wavelength, contact time, pH, and water matrix (Lu et al. 2022). RCS are short-lived and more selective than ∙OH, and they are less scavenged by NOM than ∙OH. Although both radicals are strong oxidants and contribute to indirect disinfection, the role of Cl∙ is less understood compared to ∙OH (Fang et al. 2014; Guo et al. 2022). The levels of RCS generation during chlorine-UV disinfection have not been clearly reported, specifically at varying concentrations of free chlorine species exposed to UV irradiation. Further studies are needed to optimize chlorine and UV doses, along with contact times, to maximize the generation of reactive species, improve the disinfection of emerging pathogens and ARGs, and control the DBP formation in water treatment.

#### Chlorine/UV disinfection (chlorine/UV)

This CUV-HD approach is emerging as an alternative advanced oxidation process (AOP), with a diverse role to remove the microbial contaminants as well as other emerging pollutants through the generation of ROS (∙OH, ∙O^2−^, and ^1^O_2_) and RCS (Cl∙, Cl_2_∙-, and ClO∙) (Fang et al. 2014; Guo et al. 2022). The simultaneous application of chlorine and UV, as an AOP, can generate more reactive species with more oxidation potential than standalone chlorine and UV disinfection. In this strategy, presented in Fig. 3, key mechanisms involve the generation of ROS (∙OH**,** ∙O^2−^) and RCS (Cl∙, Cl_2_∙-) by UV photolysis of free chlorine (Watts and Linden 2007; Fang et al. 2014). The rate of ∙OH generation is around two times higher than in the conventional AOP (H_2_O_2_/UV) at 254 nm UV. However, ∙OH generation is affected by UV wavelength and water pH. The HOCl/OCl^−^ ratio shifts with water pH, and a greater HOCl species favours the high ∙OH species for oxidation (Watts and Linden 2007; Guo et al. 2022). The formed Cl∙ reacts with the chloride ion (Cl∙) and forms the dichloride radical anion (Cl_2_∙^–^). The availability of the broad-spectrum oxidant (OH**·**) and selective oxidants (Cl∙, Cl_2_∙^–^) makes this hybrid approach to offer maximum synergistic effects during the water disinfection (Watts and Linden 2007; Fang et al. 2014; Guo et al. 2022). This strategy is best reported for their broader pollutants removal, less influenced by the water matrix, and better energy efficiency (Guo et al. 2022). However, water containing dissolved organic matter (DOM) and ammonia as highly reactive nitrogen species is reported to be produced (Fang et al. 2014; Moore et al. 2024).

Among the three disinfection strategies, the chlorine/UV process may generate the highest levels of reactive species (∙OH, Cl∙, Cl_2_∙-, and ClO∙), since free chlorine species (HOCl/OCl⁻) are present at relatively high concentrations and can produce additional radicals beyond free chlorine itself (Fang et al. 2014; Ma et al. 2025). In contrast, in the chlorine-UV process, the concentration of free chlorine species may be reduced or depleted before irradiation, as they are consumed through reactions with organics and during disinfection; in this case, UV light primarily targets dominantly produced chlorinated species such as chloroform and chloro-di bromo methane (Verma et al. 2017; Lei et al. 2021). In the third UV-chlorine disinfection, ∙OH is first generated through water photolysis before chlorination, and the subsequent disinfection is primarily enhanced by the resulting free chlorine species and is majorly accounted solely for direct disinfection.

The availability of free chlorine species strongly depends on water pH and matrix composition, while their decomposition is influenced by factors such as the initial chlorine dose, water pH, UV fluence, and the complexity of the water matrix (Feng et al. 2007; Hong et al. 2019). In chlorine-UV disinfection, residual free chlorine species (HOCl/OCl⁻) exposed to UV_254_ photolysis can produce both ROS and RCS, as reported in the chlorine/UV strategy (Guo et al. 2022). These primary products can then undergo secondary reactions to generate additional reactive species that amplify the disinfection process (Hong et al. 2019). However, under conditions of complex water matrices or limited free chlorine, ROS production may remain at non-lethal levels, thereby reducing disinfection efficacy against a broad range of microbial pathogens, including ARB and ARGs.

Recent evidence suggests that secondary ROS production can evolve into a self-amplifying process, leading to extensive cellular injury comparable to the terminal oxidative stress observed during antimicrobial exposure (Hong et al. 2019). Nevertheless, the mechanistic link between the initial primary damage and subsequent ROS accumulation remains unclear, and, to date, no systematic studies have addressed this phenomenon in the context of water disinfection.

## Disinfection efficacy of hybrid strategies for removal of emerging microbial pollutants

### Antibiotic resistance genes (ARGs)

ARGs removal efficiency by CUV-HD approaches follows the trend: chlorine/UV > chlorine–UV > UV–chlorine, as reported in the studies summarized in Table 1. This order of ARGs removal is supported by the generation of ROS (∙OH) and RCS (Cl∙ and Cl_2_∙–), which together promote indirect disinfection (Liu and Hu 2020; Phattarapattamawong et al. 2021; Wang et al. 2024; Zhang et al. 2024). The major ARG classes evaluated under CUV-HD include tetracycline resistance genes (*tetA*, *tetM*, *tetX*, *tetG*, *tet*(*A*)), sulfonamide resistance genes (*sul1*, *sul2*), macrolide resistance genes (*ermB*, *mph*(*A*)), β-lactam resistance genes (*blaTEM*, *bla-TEM1*, *blaCTX-M1*, *blaNDM-1*), along with the MGE (*intI1*) and the housekeeping gene (*16S rRNA*). The most reported ARGs were those conferring resistance to tetracyclines (*tetA*), sulfonamides (*sul1*), and β-lactams (*blaTEM*).

**Table 1:** Microbial pollutants removal efficacy of CUV-HD approaches in water disinfection

Target (pathogens/ARGs)	Method of quantification	Doses of disinfectants	Potential role of hybrid disinfection	Limitation	Reference
**Chlorine + UV (chlorine followed by UV) disinfection technologies**					
TRB, AmRB, MRB, SRB, and BRB and respective ARGs	Media plating and qPCR	Chlorine: 1–4 mg/L UV: 15.00–45.00 mJ/cm^−2^	Effective against BRB and TRB ARGs Bacteroidetes abundance was the lowest	Highest health risk index of ARGs after the Cl_2_-UV process	(Zhang et al. 2025)
Gram-positive: *Staphylococcus aureus* and *Bacillus subtilis* Gram-negative: *Escherichia coli*	Media plating	Chlorine: 5 mg/L UV: 126 mJ/cm^−2^	Most effective against *Escherichia coli* as compared to standalone chlorine and UV disinfection	Showed least synergistic effect as compared to UV- Cl_2_ and UV/Cl_2_ and therefore least effective against *Staphylococcus aureus* and *Bacillus subtilis* spores No DBPs were studied	(Chen et al. 2023)
Adenovirus Type 5	Plaque assay	Chlorine: up to 0.15 mg/L UV: up to 50 mJ/cm^−2^	Chlorine-UV sequence showed synergy, resulting in reduced microbial attachment and consequently decreased infection efficiency	NO DBPs were studied	(Rattanakul et al. 2015)
Coliforms	Metagenomic and media plating	Chlorine: 0.5 to 3.0 mg/L UV: 14 to 41 mJ/cm^−2^	Effectively reduced the coliforms and lower the DBPs	Increase in ARGs, *Pseudomonas*, and *Aeromonas* could not reduce effectively	(Shekhawat et al. 2021)
Coliphages and Enterobacteria phage MS2	Plaque assay	Chlorine: 0.05–0.25 mg/L UV:14–22 mWs/cm^−2^	Combined treatment achieved 3–5 log₁₀ reductions of chlorine-resistant strains	No DBPs were studied	(Zyara et al. 2016)
Coliforms	Media plating and qPCR	Chlorine: 0.5 mg/L UV: 14–55 mJ/cm^−2^	Effectively suppressed the post-disinfection regrowth	No DBPs were studied	(Shekhawat et al. 2023)
**UV + chlorine (UV followed by chlorine) disinfection technologies**					
ARGs (*sul1*, *tetX*, *tetG*, and *intI1)* in municipal wastewater	qPCR	UV: 9.85 mW/cm^−2^ Chlorine: 5–30 mg/L	Hybrid disinfection was more effective in ARGs removal than standalone chlorine and UV irradiation	Ammonical nitrogen reduces the efficacy of chlorination with UV doses (62.4 mJ/cm^−2^, 124.8 mJ/cm^−2^, and 249.5 mJ/cm^−2^) and high chlorine (5 to 30 mg/L)	(Zhang et al. 2015)
OPs: *Aeromonas* spp., *Legionella* spp., *Mycobacterium* spp., *Pseudomonas aeruginosa*, *Legionella pneumophila*, *Mycobacterium avium*, *Mycobacterium fortuitum*, and post-disinfection regrowth	qPCR, Illumina HiSeq analysis	UV: 40 mJ/cm^−2^ Chlorine: 1.55—1.59 mg/L	Combined disinfection synergistically controls the OPs or likely due to the ecological interaction and microbial community structure changed by UV/Cl_2_	OPs residing in biofilms are tolerant to UV + chlorine disinfection	(Liu et al. 2019)
TRB, AmRB, MRB, SRB, and BRB and respective ARGs	Media plating and qPCR	UV: 15.00—45.00 mJ cm^⁻2^ Chlorine: 1–4 mg/L	Health risk index of ARGs after the UV-Cl_2 _process was relatively low	Lesser ARG removal efficiency than simultaneous Cl_2_/UV disinfection	(Zhang et al. 2025)
HPC, TBC, and TC	Media plating	UV 4–40 mJ/cm^−2^ Chlorine: 1.6–4.5 mg/L	After UV treatment (40 mJ/cm^−2^) followed by chlorine treatment (2.0 mg/L), TBC and HPC were reduced by 3.46 and 4.50 log units, respectively No observable photoreactivation of bacteria Prior UV sensitization relatively reduces TTHM as compared to standalone chlorine	Only UV followed by chlorine is investigated, but not reverse	(Wang et al. 2012)
*Staphylococcus aureus*, *Bacillus subtilis*, *Escherichia coli*	Media plating	UV: 126 mJ/cm^−2^ Chlorine: 5 mg/L	Highest synergistic efficiency as compared to Cl_2_/UV and Cl_2_-UV and therefore inhibited the repair process of *Bacillus subtilis* spores (most resistant)	No DBPs were studied	(Chen et al. 2023)
Sulphonamide resistant gene *sul*1	qPCR	UV: 24–288 mJ/cm^−2^ Chlorine: 20 mg/L	UV attacks on large DNA fragments and chlorine on smaller fragments Major contribution of HO· in sul1 gene removal	No DBPs were studied	(Liu and Hu 2020)
Bacteriophage surrogates (MS2 and PR772)	Plaque assay	UV: 20–40 cm^−2^ Chlorine: 0.3–3.5 mg/L	Synergistic effect where UV damages the MS2 protein and enhances the sensitivity for chlorine	No DBPs were studied	(Gao et al. 2023)
*Escherichia coli*; *Shigella dysenteriae*	Media plating	UV: 8 mJ cm^−2^ Chlorine: 1.5 mg/L	Suppression of post-disinfection regrowth	No DBPs were studied	(Wang et al. 2012)
QNs ARGs: *acra-01*, *flor*; β-LACs ARGs: *ampC1*, *blaCTX-M1*, *blaTEM* MLs ARGs: *ermA*, *ermB*, *ermC*, *ermF* AGs ARGs: *strA* SAs ARGs: *sul1*, *sul2* TCs ARGs: *tetB*, *tetC*, *tetM*, *tetO*, *tetQ*, *tetW*	qPCR	UV: 15–100 mJ/cm^2^ Chlorine: 10—30 mg/L	UV (15 mJ/cm^2^) and chlorine (10 mg/L) combination achieved 1.46 log ARGs removal, which was 0.73 log higher than standalone UV (100 mJ/cm^−2^) or chlorine (30 mg/L)	No DBPs were studied	(Wei et al. 2024)
Adenovirus Type 5	Plaque assay	UV: up to 50 mJ/cm^−2^ Chlorine: up to 0.15 mg/L	4 log inactivation of HAdV-5	Synergy was absent No DBPs were studied	(Rattanakul et al. 2015)
TET-RB, AMP-RB, CHL-RB, and STR-RB	Media plating	UV: up to 80 mJ/cm^−2^ Chlorine: up to 30 mg/L	Reduction in potential pathogenic bacteria, produces fewer DBPs, prevents mitigating the reactivation of ARB, and also demonstrates a synergistic effect	Mobile genetic elements could not reduce photoreactivation and dark repair ability of AMP-RB increases after sequential disinfection compared to UV disinfection	(Cheng et al. 2024)
UV-resistant bacteria *Deinococcus soli* *Sphingomonas panni*	Media plating and qPCR	UV: 0–40 mJ/cm^−2^ Chlorine: 0.5–1.0 mg/L	Synergism obtained Cytoplasmic degradation used for bacterial antioxidant property loss	No DBPs were studied	(Cao et al. 2025)
Faecal coliforms	Media plating	UV: 24 mJ/cm^−2^ Chlorine: 0.5–5 mg/L	High oxidants enhanced the faecal coliform inactivation	Reactivation rate of faecal coliform is high than simultaneous application of UV and chlorine No DBPs were studied	(Lu et al. 2022)
ARB: ARGs-*tet(A)*, *bla-TEM1*, *sul1*, and mph(A)	qPCR and media plating	UV: 50–200 mJ/cm^−2^, chlorine: 1–2 mg/L	< 2 log reduction of ARGs and reduction order of ARGs bla-TEM1 > mph(A) > tet(A) > sul1, at UV 200 mJ/cm^−2^ + chlorine 2 mg/L	No DBPs were studied	(Destiani and Templeton, 2019)
**Simultaneous chlorine/UV disinfection technologies**					
ARB: *Morganella morganii* *Enterococcus faecalis* ARGs: *aacC2* *sul2* *strB* *tetA* *tetB* RP4 plasmid conjugation	qPCR and media plate counts	Chlorine: ≥ 1 mg/L UV: ≥ 4 mJ/cm^−2^	Risk of RP4 plasmid conjugation transfer was significantly reduced Reduction in post-disinfection regrowth Inhibited the photo reactivation of ARB	No chlorine followed by UV disinfection was performed No DBPs was studied	(Wang et al. 2020)
Opportunistic pathogen: *Staphylococcus aureus*	Media plate	Chlorine: 2 mg/L UV: 18 mJ/cm^−2^	7 log reduction or no detectable *Staphylococcus aureus* was found Inhibited post-disinfection regrowth	No DBP study was conducted	(Chen et al. 2022)
Pathogens: *Pseudomonas aeruginosa* *Staphylococcus aureus* *Escherichia coli* *Salmonella* spp., *Legionella pneumophila*, *Shigella* spp., *Yersinia*, *Vibrio cholerae*, *Mycobacterium avium* ARGs and bacterial communities	qPCR and high-throughput sequencing	Chlorine: upto 6 mg/L UV: 0.2 mW/cm^−2^ for up to 30 min	Highly effective against *L. pneumophila*, *Mycobacterium avium* in all four seasons Most ARGs were reduced except some antibiotic ARGs aminoglycosides: aac(6′)-II and aacA_aphD, three tetracyclines: tetA-02, tetPB-03, and tetR-02; three multidrugs: mexF, qacEdelta1-01, and qacEdelta1-02; and three MGEs: intI-1(clinic), intI-1LC, and tnpA-05 were consistent in all the seasons	Less effective against *Pseudomonas aeruginosa*, *Staphylococcus aureus*, *Shigella* spp*.*, and *Yersinia*, especially in winter and autumn season collected samples *Acinetobacter* and *Mycobacterium* were the main bacterial genera in the community and both are potential hosts for tnpA gene (MGEs) No DBPs were studied	(Ye et al. 2021)
TRB, AmRB, and ARGs -*tetM* and *blaTem*	qPCR and media plating	Chlorine: 0.5 to 20.0 mg/L UV: 3.59–9.03 mW/cm^−2^	Highly effective for the removal of tetM (0.98–3.20 log) and blaTem (1.28–3.36 log)	Requires high chlorine doses No DBPs were studied	(Phattarapattamawong et al. 2021)
ARB, TRB, AmRB, MRB, SRB, and BRB and respective ARGs	Media plate and qPCR	Chlorine: 1.00–4.00 mg/L UV: 15.00–45.00 mJ/cm^−2^	Simultaneous UV/chlorine approach found effective in reduction of ARGs as compared to sequential UV-chlorine or vice versa	Less effective than the sequential disinfection in inactivating BRB and SRB No DBPs were studied	(Zhang et al. 2025)
ARB and ARGs	Media plate and qPCR	Chlorine: 55‒300 mg/L UV: 0.87 mW/cm^−2^	*ARGs (tet*A, *sul*1, *sul*2, and *erm*B) degraded in smaller fragments Risks of HGT were reduced Control in DBPs production	No wastewater samples were studied	(Zhang et al. 2024)
Faecal coliforms	Media plating	Chlorine: 0.5—5 mg/L UV: 24 mJ/cm^−2^	Suppresses the reactivation rate of faecal coliform post disinfected samples	No DBPs were studied	(Lu et al. 2022)
*Staphylococcus aureus* *Bacillus subtilis* *Escherichia coli*	Media plating	Chlorine: 0–2 mg/L UV: 9 mJ/cm^−2^	Complete inactivation of *S. aureus*	No DBPs were studied Less effective for *Bacillus subtilis* spores	(Chen et al. 2023)
ARGs and *Escherichia coli* HB101	qPCR, media plating, and flow cytometry	Chlorine: 3.5 mg/L UV: 0.54 ± 0.03 mW/cm^−2^	Effective in removing ARGs Damages the cell membrane due to RCS	No DBPs were studied	(Wang et al. 2024)
Sulphonamide resistant gene *sul*1	qPCR	Chlorine: 20 mg/L UV: 24 to 288 mJ/cm^−2^	Major contribution of HO· in *sul* 1 gene removal	No DBPs were studied	(Liu and Hu 2020)
*Pseudomonas aeruginosa*, *opr* gene	Media plating, qPCR	Chlorine:1 mg/L UV: 0.2 mW/cm^−2^	Major contribution of RCS Effective in post-disinfection regrowth suppression *opr* gene removal	No DBP were studied	(Wang et al. 2021)
Antibiotic resistant bacteria: blaNDM-1-carrying *Escherichia coli* Sulphonamide-resistant *Escherichia coli* TOP 10F	Media plating and qPCR	Chlorine: 5 mg/L UV: 10 W exposed up to 30 min	UV/NaClO disinfection reduced intracellular (4.5–5.7 log) and extracellular (2.3–3.4 log) ARGs achieved 3.3 log *intI* gene removal via ROS and RCS Effectively inhibited plasmid-borne blaNDM-1 transfer	No DBPs were studied	(Ma et al. 2025)
UV-resistant bacteria *Deinococcus soli* *Sphingomonas panni*	Media plating and qPCR	Chlorine: 0.5–1.0 mg/L UV: up to 40 mJ/cm^−2^	Synergism obtained Cytoplasmic degradation used for bacterial antioxidant property loss	No DBPs were studied	(Cao et al. 2025)

*AMP-RB* ampicillin-resistant bacteria, *AmRB* aminoglycoside-resistant bacteria, *BRB* β-lactam-resistant bacteria, *blaTem* β-lactamase gene, *CHL-RB* chloramphenicol-resistant bacteria, *DBP* disinfection byproducts, *HPC* heterotrophic plate count, *MLs* macrolides, *OPs* opportunistic pathogens, *QNs* quinolones, *RCS* reactive chlorine species, *ROS* reactive oxygen species, *SRB* sulfonamide-resistant bacteria, *STR-RB* streptomycin-resistant bacteria, *TBC* total bacteria count, *TC* total coliform, *TTHM* total trihalomethanes, *UV* ultraviolet light

During chlorine/UV disinfection, generated ∙OH shows a greater selectivity towards reacting with and degrading nitrogenous bases compared to other organic radicals produced in other hybrid strategies (UV/H₂O₂ and UV/PAA) (Wang et al. 2024). In contrast, studies investigating *blaNDM-1* under chlorine/UV disinfection in both intracellular (iARG) and extracellular (eARG) forms observed that intracellular *blaNDM-1* was removed more effectively (> 4.0 log) than extracellular *blaNDM-1* (< 4.0 log) within a 30-min contact time. However, the presence of DOM in water inhibited the removal efficiency of iARGs more strongly than eARGs (Ma et al. 2025). Therefore, higher eARG removal may be achieved using chlorine/UV disinfection. This may also suggest that cellular damage caused by chlorine/UV leads to the release of iARGs into the water, increasing eARG levels and contributing to their higher apparent removal efficiency (Wang et al. 2024; Ma et al. 2025). In another study, both iARGs and eARGs, including *sul1*, *sul2*, *tetA*, and *ermB*, were examined. The initial copy numbers of iARGs (∼2 × 10⁸ copies/mL) were substantially higher than eARGs (∼10^3^ copies/mL). Following treatment, iARGs (*sul2*, *tetA*, and *ermB*) showed high removal efficiencies (> 6.0 log), whereas *sul1* exhibited slightly lower removal (between 4.0 and 5.0 log). In contrast, all eARGs (*sul1*, *sul2*, *tetA*, and *ermB*) were reduced to below the detection limit (Zhang et al. 2024).

Many studies highlighted the major role of ROS, specially ∙OH and RCS, specifically Cl∙ and Cl_2_∙^−^ in ARGs removal (Liu and Hu 2020; Chen et al. 2022; Zhang et al. 2024, 2025). Compared to other microbial pollutants, ARGs are more resistant and less susceptible to degradation (Wang et al. 2020; Phattarapattamawong et al. 2021). ARGs such as *tet*M and *bla*TEM were effectively removed (> 3.00 log) by the chlorine/UV process, whereas ∙OH plays the dominant role, while RCS contributes to a lesser extent. However, *bla*TEM has shown more sensitivity than *tet*M (Phattarapattamawong et al. 2021) and a similar kind of data was reported by Destiani and Templeton (2019). Moreover, the *blaTem* gene was found to be least tolerant compared to other genes (*tetA*, *mphA*, *sul*1). The generation of ∙OH significantly enhanced the removal of tetM and *blaTEM* by 48 and 19%, respectively. The relatively lower contribution of ∙OH to *blaTEM* removal can be attributed to its high competition with chlorine during the reaction. Additionally, different ARGs appear to vary in their sensitivity to ∙OH. Among the studied genes, the UV/chlorine process was most effective for *blaTEM* removal, followed by *tetA* and *sul1* (Destiani and Templeton, 2019; Ma et al. 2025). This strategy has been shown to generate more reactive species compared to other hybrid disinfection methods (Phattarapattamawong et al. 2021; Zhang et al. 2024; Ye et al. 2021). ARG removal was closely correlated with the removal of MGEs, although the removal trend for specific ARGs remained similar and largely non-selective. Nevertheless, ARGs containing a high proportion of MGEs were found to be more persistent and resistant (Ye et al. 2021).

In UV-chlorine strategy, ARGs were also shown to be effectively reduced and the process suppressed the photo-repair of sulfonamide and tetracycline resistance genes more efficiently than standalone UV disinfection. However, β-lactam resistance genes (*blaTEM*, *blaCTX-M1*) were not effectively reduced by UV-chlorine disinfection, suggesting lower sensitivity to chlorine (10 mg/L) compared to UV irradiation (9 mJ/cm^2^) (Wei et al. 2024). Further supported by Zhang et al. (2015) conducted for removal efficacy of ARGs (*sul1*, *tetX*, and *tetG*), MGE (*intI1*), and the housekeeping gene (*16S rRNA*), it showed that removal increases with increasing UV exposure (62.4 to 249.5 mJ cm^− 2^) in the UV-chlorine strategy. However, water containing higher ammonia (15 mg/L) affected the ARGs’ removal by 80%.

All three CUV-HD strategies against 28 ARGs of six different classes reported the chlorine/UV more effective than sequential disinfection approaches for all the ARGs, and it is noticed that chlorine/UV is two times more effective than chlorine-UV and UV-chlorine against sulfonamide resistance genes and especially for *sul1* reduction (Zhang et al. 2025). Its high removal efficiency was attributed to the combined action of ∙OH and RCS, as well as the higher G + C content of certain ARGs, which makes them more susceptible to radical attack. Since UV irradiation itself is equally reactive towards all DNA bases, ARGs with higher G + C content are more efficiently removed when higher UV doses are applied (Zhang et al. 2025). The removal efficiency of ARGs is influenced by their initial abundance, with high-copy-number genes being more readily eliminated, while low-copy-number ARGs tend to exhibit greater resistance to disinfection. For example, ARGs linked with multidrug efflux pumps, which typically occur in low abundance, were found to be the most resistant across all strategies (Zhang et al. 2025). When compared to conventional AOPs such as UV/H₂O₂ and UV/PAA, chlorine/UV disinfection achieved better removal of ARGs. In this case, RCS (Cl∙, Cl_2_∙-, and ClO∙) played the dominant role in ARG degradation, despite the relatively low production of ∙OH compared to UV/H₂O₂. This further indicates the critical contribution of RCS in this process (Liu and Hu 2020). However, studies focusing on the *sul*1 demonstrated that chlorine/UV disinfection achieved synergistic removal, primarily due to ∙OH activity, while Cl∙ showed negligible involvement. Although other RCS species were not fully investigated (Liu and Hu 2020). Further studies based on reactive species such as ∙OH, Cl∙, Cl_2_∙-, and ClO∙ may provide a better understanding of the ARG degradation mechanism in water.

### Antimicrobial/antibiotic resistance bacteria and opportunistic pathogens

Many studies similarly reported that the chlorine/UV strategy is most effective for the removal of ARB and presented in Table 2 (Chen et al. 2022; Ma et al. 2025). However, increasing doses of chlorine or UV enhanced the ARB removal efficiency in either hybrid strategy (Zhang et al. 2025). In a study, it was reported that β-lactam-resistant bacteria were more prominent as compared to other ARB such as aminoglycoside-resistant bacteria, macrolide-resistant bacteria, sulphonamide-resistant bacteria, and tetracycline-resistant bacteria. The sulfonamide-resistant bacteria and aminoglycoside-resistant bacteria were reported to pose a challenge for the hybrid disinfection approaches. Similarly, sulfonamide resistance genes that were previously reported have been observed with the highest resistance for all presented hybrid disinfection approaches. A high concentration of chlorine (60 mg/L) is needed to control sulfonamide-resistant bacteria in wastewater; even at low UV doses (20.00 mJ cm^−2^), the percentage of such ARB increases in the final effluent (Zheng et al. 2017). In a combined disinfection approach, increasing the UV dose up to 45 mJ cm^−2^ increases the removal rate of ARBs (Zhang et al. 2025). Ma et al. (2025) demonstrated the high efficacy of chlorine/UV disinfection for the removal of ARGs, antibiotics, and ARBs. ARBs harbouring the *bla*_NDM-1_ gene have been effectively removed (7 log reduction) in reclaimed water without being influenced by the water matrix. The removal mechanisms have been underscoring the role of reactive species such as ∙OH and ∙Cl in severe bacterial membrane damage, promoting bacterial lysis and release of cytoplasmic content and thereby preventing post-disinfection regrowth. However, other sequential strategies such as chlorine-UV and UV-chlorine were not studied. The hybrid disinfection strategy, UV-chlorine, has reported a higher potential for the reduction of coliforms as well as their post-disinfection regrowth as compared to standalone disinfection, to meet the coliform regulatory requirements for wastewater reuse practices (Gupta et al. 2022). The assessment of three hybrid disinfection approaches (chlorine-UV, UV-chlorine, and chlorine/UV) against Gram-negative (*E. coli*) and Gram-positive bacteria (*Staphylococcus aureus* and *Bacillus subtilis spores*) showed a synergistic effect of the chlorine/UV approach for the removal of highly disinfection-resistant bacteria (*Staphylococcus aureus* and *Bacillus subtilis* spores), and a dose of UV (9 mJ/cm^2^) and chlorine (2 mg-Cl/L) inactivated *S. aureus* completely. However, sequential hybrid disinfection approaches were effective in inactivating *E. coli* at lower doses but did not show a synergistic effect for *E. coli* (Chen et al. 2023). In another study, an opportunistic pathogen, *Pseudomonas aeruginosa*, was irreversibly eliminated by the chlorine/UV disinfection approach, though culturable elimination has been observed with standalone chlorine and UV; however, they were unable to remove the viable but non-culturable cells (VNBC). RCS (Cl∙, Cl_2_∙⁻, and ClO∙) generated during chlorine/UV disinfection which played a critical role in the irreversible damage of *P. aeruginosa*. Moreover, this approach promoted a reduction of the metabolic rate and loss of the toxic *opr* gene by 99% (Wang et al. 2021). However, sequential chlorine and UV disinfection have not been studied to compare the efficacy of all combined chlorine and UV disinfection strategies. Other studies similarly reported the control of OP regrowth post-hybrid disinfection, including chlorine/UV and UV-chlorine; therefore, it is indicated that residual chlorine is crucial to control microbial regrowth in hybrid disinfection strategies (Liu et al. 2019; Shekhawat et al. 2023). Hybrid disinfection approaches have been studied for inactivating viruses resistant to chlorine, such as bacteriophage MS2, and resistant to UV, such as human adenovirus 5 (HAdV-5), in water. Sequential disinfection approaches have shown a synergistic impact in the reduction of bacteriophage MS2 and HAdV-5 (Rattanakul et al. 2014, 2015; Gao et al. 2023). The chlorine-UV disinfection approach has shown more potential as compared to the chlorine/UV approach to eliminate bacteriophage MS2 and HAdV-5. The initial chlorine treatment supported the viral capsid damages, and the following UV disinfection effectively inactivated MS2 and HAdV-5. UV-chlorine disinfection effectively damaged the genome of viruses, and following chlorine exposure enhanced their inactivation. However, HAdV-5 may repair, regrow, and be able to infect the host cells. Therefore, in viral disinfection, pre-chlorine or UV sensitization has a potential impact to increase the removal efficacy of hybrid disinfection approaches. UV and chlorine-resistant bacteria isolated from wastewater were studied for sensitivity against the chlorine/UV and UV-chlorine disinfection strategies. Similarly, *Deinococcus soli* has reportedly been synergistically removed by both the applied disinfection processes; however, *Sphingomonas panni* reported a synergistic removal synergistically by the chlorine/UV disinfection approach (Cao et al. 2025). Thus, discussed studies have indicated that the chlorine/UV disinfection strategy shows enhanced removal efficacy of microbial pathogens as compared to sequential disinfection approaches (chlorine-UV and UV-chlorine).

**Table 2:** Comparison of CUV-HD approaches for wastewater disinfection

Biological contaminants	Chlorine-UV sequential	UV-chlorine sequential	UV/chlorine (simultaneous)
Bacteria	Lower synergy but effective against many chlorine and UV resistant bacteria. No residual chlorine	Moderate but effective against chlorine and UV resistant bacteria and residual chlorine effectively reported to post-disinfection regrowth	Highest bacterial inactivation due to simultaneous ROS and RCS (∙OH, Cl∙, ClO∙) generation. Effective even for UV/chlorine-resistant strains
ARGs	More effective than UV-chlorine due to higher reactive species than UV-chlorine	Least effective than other two CUV-HD strategies	Strongest ARG degradation. Radical-driven oxidation fragments both intra- and extracellular ARGs
Opportunistic pathogens) (OPs)	Improved compared to single disinfectants, but often less efficient than UV-chlorine	Higher efficiency for pathogens (e.g., *P. aeruginosa*)	Most efficient; simultaneous attack by UV + radicals achieves rapid pathogen removal
Viruses	Adenovirus and MS2 best controlled than in UV-chlorine or simultaneous	More effective for adenovirus than chlorine-UV; UV damages viral capsids before chlorine attack	synergy reported for MS2 but not for adenovirus
DBPs formation	Lowest DBP formation (as chlorinated DBPs are targeted with post UV exposure)	Higher DBP potential than chlorine-UV but still less than chlorine/UV	Highest DBP formation due to radical-induced transformation of NOM and chlorine
Influence of water matrix (NOM, suspended solids (SS), turbidity)	Chlorine can reduce microbial shielding before UV, but UV penetration still limited by turbidity	NOM consumes chlorine radicals after UV exposure; efficiency depends on water quality	Least affected but NOM and suspended solids influence; radical scavenging reduces efficiency and raises DBP risks
Synergy level	Low synergy	Moderate synergy	High synergy reported
Best uses	Municipal sewage/reclaimed water where DBP minimization is important	Applications prioritizing high ARG and pathogen removal with balanced DBP risk	Situations demanding maximum disinfection and ARG control, but with attention to DBP risk

### Disinfection byproducts (DBPs)

Apart from microbial contaminant removal, controlling the generation of DBPs during disinfection processes is a major challenge (Liu et al. 2019; Forster et al. 2025). DBPs are regarded as emerging pollutants and can be treated as a separate class of contaminants like microplastics or pharmaceuticals. Chlorine, as one of the most widely used water and wastewater disinfectants, raised concern when J. J. Rook first identified chloroform as a DBP in chlorinated water in 1974. Subsequently, many studies reported different types of DBPs in chlorinated water, highlighting their potential health concerns (Du et al. 2024; Forster et al. 2025). The relatively high concentrations of DBPs, such as trihalomethanes (THMs) and haloacetic acids (HAAs), in chlorinated water led to the development of policy frameworks to regulate their limits (Sinha et al. 2021; Villanueva et al. 2023). In chlorinated water, the most commonly reported THMs are chloroform, bromoform, di-bromochloromethane, and di-chloro bromomethane, and the HAAs are chloroacetic acid, di-chloro acetic acid, and bromo acetic acid from chlorinated water (Verma et al. 2017; Sinha et al. 2021).

Over time, more than 700 DBPs have been identified, while a few are known to be toxic; many remain poorly understood (Richardson and Kimura 2019). Hybrid disinfection strategies have been investigated to control DBPs’ formation in treated water (Liu et al. 2019; Du et al. 2024). The primary goal of reducing the chlorine dose in these strategies is to limit DBP generation while taking advantage of the synergistic effect for enhanced removal of microbial pollutants. NOM and its reactivity with chlorine and the reactive species it produces, including ROS and RCS, play a key role in DBP formation across different disinfection approaches.

A variety of DBP precursors have been identified. Among them, the unsaturated and aromatic fractions of NOM, especially humic acids and fulvic substances, are the most commonly reported contributors to THMs and HAAs (Hua and Reckhow 2007; Xiao et al. 2024; Forster et al. 2025). Other precursors include microbial-derived amino acids, peptides, and proteins, as well as anthropogenic contaminants such as agricultural discharges and pharmaceutical products, all of which can lead to the formation of different DBPs (Shen and Andrews 2011).

In chlorine/UV disinfection, many studies reported that DBPs’ formation increases due to higher concentrations of generated ROS and RCS, giving the process a greater potential to form different classes of DBPs (Hua et al. 2021; Wang et al. 2023). Compared to chlorination alone, a short contact time of chlorine/UV treatment has been reported to increase the formation of DBPs such as THMs, HAAs, haloacetonitriles (HANs), and others by 90–508% while also increasing cytotoxicity and genotoxicity (Hua et al. 2021). In another study, generation of DBPs from NOM during post-chlorine disinfection by UV/chlorine increased by 10–50% (Wang et al. 2023).

In UV-chlorine disinfection, prior application of UV promotes the photooxidation of humic and fulvic acids, reducing the formation of THMs and HAAs (Hua and Reckhow 2007; Shen and Andrews 2011). UV pretreatment also breaks down pharmaceuticals and amine-based precursors, thereby lowering DBP yields. In addition, UV can degrade nitrogenous DBPs, such as NDMA (N-nitrosodimethylamine), as well as other DBPs like chloroform and bromoform, further reducing DBP formation (Shen and Andrews 2011). However, UV photo-oxidized precursors can break down into smaller, more reactive compounds, which have been reported to increase the formation of DBPs, including nitrogenous DBPs (Liu et al. 2021). In another approach, chlorine-UV disinfection, chlorine reacts with different precursors in water to form DBPs. These produced DBPs can then be further degraded by subsequent UV exposure, which has been reported to reduce DBP formation by targeting THMs and HAAs (Hua and Reckhow 2007; Liu et al. 2021). However, UV exposure, along with available chlorine, may increase the formation of DBPs due to generated reactive species. Conversely, applying UV after free chlorine has been exhausted has been reported to enhance the removal of DBPs formed during chlorination via photolysis, highlighting the importance of post-UV treatment to limit DBP formation. Therefore, post-UV disinfection with UV-chlorine and chlorine/UV may be a potential approach for the overall safety of treated wastewater reuse practices. From previous reported studies, the DBP formation potential among different hybrid disinfection strategies can be summarized as UV/chlorine > UV-chlorine > chlorine-UV. This order of DBP formation potential may support the generation of ROS and RCS in different CUV-HD approaches. However, mechanistic insights into the reactions of many precursors remain limited, and dose optimization of UV and chlorine, and the subsequent interactions of photolyzed molecules with chlorine strongly influence DBP formation.

## Conclusions and future prospectives

The CUV-HD strategies present a high potential to overcome the limitations (high chlorine doses, post-disinfection regrowth, chlorine/UV tolerant bacteria) raised by the standalone chlorine and UV disinfection. A classification of disinfection technologies is presented to facilitate a systematic understanding of their mechanisms, roles, and research challenges. Most studies summarized in Table 1 report chlorine doses between 0.5 and 2.00 mg/L, and UV dose ranges between 10 and 80 mJ cm^−2^ have been shown to achieve the goal of the reduction of chlorine doses. The ARG removal efficiency for the CUV-HD approaches follows the trend: chlorine/UV > chlorine–UV > UV–chlorine, while DBP formation potential order is indicated as UV/chlorine > UV-chlorine > chlorine-UV. The chlorine/UV hybrid disinfection strategy has indicated enhanced overall disinfection efficacy against microbial pollutants by exploiting the oxidizing capacity of reactive species such as ∙OH, ∙O₂⁻, Cl∙, Cl_2_∙⁻, and ClO∙. The role RCS in the formation of various DBPs in the treated wastewater is a potential challenge for the chlorine/UV disinfection strategy. Chlorine–UV disinfection has been reported to produce lower levels of DBPs and has the potential to be adopted as a safer disinfection strategy. However, further optimization (chlorine and UV dose and contact time) of this approach is needed to enhance its disinfection efficacy. Beyond reducing chlorine doses, the focus of CUV-HD research is shifting towards exploiting the reactive species generated during the process, which primarily support indirect disinfection mechanisms. Despite increasing investigations into CUV-HD strategies, very few studies have quantified reactive oxygen species (ROS) and reactive chlorine species (RCS) to clarify their exact role in the indirect disinfection of ARGs, ARB, and other emerging pollutants. Further studies are needed to focus on the generated reactive species, particularly reactive chlorine species, as their role in the disinfection process remains unclear.

a) Currently, a clear understanding of reactive species’ role in indirect disinfection of ARGs in treated wastewater remains a technical challenge due to the scarcity of expertise in assessing the concentrations and their inactivation mechanisms.

b) ROS and RCS indicate an important role in indirect disinfection. RCS (Cl∙, Cl_2_∙, and ClO∙) are more selective than ROS in targeting aromatics, which are common precursors of many DBPs, and are known to specifically target the aromatics. However, how to exploit the RCS (Cl∙, Cl_2_∙, and ClO∙), which are more specific in targeting aromatics, is a structure in certain precursors of DBPs. Therefore, exploiting the selectivity of RCS during chlorine/UV disinfection can help limit DBP formation.

c) UV disinfection has been shown to degrade the many DBPs including chloroform, bromoform, NDMA, and other aromatic DBPs. Therefore, UV exposure post chlorine/UV disinfection may control the DBPs’ formation and may fulfil the overall goal of safe reuse of treated wastewater.

## Data Availability

All data are included in this article, and the cited data are properly referenced in the manuscript.
